# A preclinical evaluation of an autologous living hyaline-like cartilaginous graft for articular cartilage repair: a pilot study

**DOI:** 10.1038/srep1622510.1038/srep16225

**Published:** 2015-11-09

**Authors:** Yvonne Peck, Pengfei He, Geetha Soujanya V. N. Chilla, Chueh Loo Poh, Dong-An Wang

**Affiliations:** 1 Division of Bioengineering, School of Chemical and Biomedical Engineering, Nanyang Technological University, 637457, Singapore

## Abstract

In this pilot study, an autologous synthetic scaffold-free construct with hyaline quality, termed living hyaline cartilaginous graft (*LhCG*), was applied for treating cartilage lesions. Implantation of autologous *LhCG* was done at load-bearing regions of the knees in skeletally mature mini-pigs for 6 months. Over the course of this study, significant radiographical improvement in *LhCG* treated sites was observed via magnetic resonance imaging. Furthermore, macroscopic repair was effected by *LhCG* at endpoint. Microscopic inspection revealed that *LhCG* engraftment restored cartilage thickness, promoted integration with surrounding native cartilage, produced abundant cartilage-specific matrix molecules, and re-established an intact superficial tangential zone. Importantly, the repair efficacy of *LhCG* was quantitatively shown to be comparable to native, unaffected cartilage in terms of biochemical composition and biomechanical properties. There were no complications related to the donor site of cartilage biopsy. Collectively, these results imply that *LhCG* engraftment may be a viable approach for articular cartilage repair.

Articular cartilage is a highly organized connective tissue with poor reparative capacity due to its low metabolic activity and avascular nature^1^. Owing to its load-bearing function, focal cartilage lesions of the knee are common and are typically located on the femoral condyles2. Inappropriate management of knee-joint related injuries may lead to serious downstream problems like osteoarthritis (OA), which is a cartilage degenerative disease that severely affects patient quality-of-life^3^. Currently, a variety of surgical techniques have been developed for treating cartilage-related injuries, including microfracture, mosaicplasty and autologous chondrocyte implantation (ACI)^4^.

Microfracture is a marrow stimulation technique that is popular as a first-line treatment for articular cartilage defects in the knee^5^. It is a reparative strategy that involves migration of MSCs from the bone marrow into the defective site. This technique usually results in fibrocartilaginous repair tissue, which is biochemically and biomechanically inferior to native hyaline cartilage^6^,^7^. Osteochondral transplantation (or mosaicplasty) is another long-standing surgical option based on the transplantation of one or more cylindrical osteochondral grafts from a minimal load-bearing region of the knee to the defects. It had demonstrated positive clinical benefits for young patients with an active lifestyle^8^. However, its use may be limited by scarce donor tissue source and donor site morbidity^8^,^9^. ACI is a cell-based approach for treating cartilage lesions^10^. This technique involves the harvesting of cartilage arthroscopically, and re-implanting culture-expanded autologous chondrocytes into a chondral defect underneath a patch of periosteum. A recent study on the long-term outcome of first generation ACI reported satisfying clinical results in terms of patient satisfaction, pain relief, and functional improvement of the knee^11^. However, it was also reported that full restoration of knee function was not achieved. On this note, a possible explanation may be the culture–expansion of cells in a 2D environment, which led to alterations in cellular phenotype, thereby compromising repair efficacy^12^,^13^. Hence, it is clear that a ‘gold standard’ for the repair of cartilage injuries is still lacking; all existing surgical interventions produce inconsistent repair quality^14^,^15^,^16^. This lack of effective treatment modalities has prompted research on tissue engineering techniques for the next generation of cartilage regeneration and repair solutions.

The tissue engineering approach offers realistic hope of restoring articular cartilage, and has since evoked widespread interest over the past decade^17^. Its principle involves combining a variety of cell sources, scaffold materials and bioactive factors to aid cartilage repair^18^,^19^,^20^. This appears to be advantageous over the direct usage of cells, which may be implicated by cell handling and delivery issues^15^. However, significant challenges remain with regards to the generation of a durable articular cartilaginous graft *in vivo*. This is largely due to a non-hyaline cartilage matrix, which contains other constituents such as type I collagen and other residual biomaterials, thereby compromising neotissue quality^17^. In order to generate cartilage matrix with hyaline-like phenotype, scaffold-free approaches are attractive as they minimize the introduction of any exogenous materials from scaffolds into the neotissue^21^. Additionally, a three-dimensional (3D) environment is crucial to prevent chondrocyte dedifferentiation, a process that causes them to lose their differentiated phenotype. In view of these requirements, a unique methodology that allows chondrocytes to form neocartilage through an intermediate 3D scaffolding system was developed^22^. The transient use of scaffolds prevents the long-term involvement of exogenous scaffold materials, thus preserving tissue purity. In addition, this adopted, non-cell adhesive 3D culture strategy hinders focal cell adhesion, thus avoiding dedifferentiation of chondrocytes induced by monolayer culture expansion^23^. The end product is a 3D scaffold-free construct known as the Living Hyaline Cartilage Graft (*LhCG*).

The prototype of *LhCG* was established previously^24^. It is constructed by culturing chondrocytes in a 3D interim scaffolding system made up of removable, non-cell adhesive micro-cavitary hydrogel (MCG)^22^. This environment promotes vigorous chondrocyte growth and abundant cartilage-specific extracellular matrix (ECM) production, hence resulting in maximal maintenance of the desired hyaline phenotype. Once the hyaline-like cartilaginous neotissue is sufficiently yielded, the scaffolding material phase is completely removed from the MCG construct. This results in a macro-scaled, sponge-like, synthetic scaffold-free product of *LhCG*, which had earlier been demonstrated to recapitulate characteristic markers of an articular cartilage *in vitro*. Furthermore, in a leporine model for articular cartilage repair, results strongly demonstrated the advantage of the *LhCG* in mediating efficacious repair *in vivo*^24^.

In this report, a preclinical study was conducted in skeletally mature mini-pigs with the eventual aim of achieving clinical translation of *LhCG*. This 6-month pilot study was designed to validate the performance of autologous *LhCG* engraftment. Skeletally mature mini-pigs were chosen as their joint sizes and cartilage thickness are amenable to clinically relevant manipulations and outcome assessments^25^. The major load-bearing regions located on the femoral condyles were selected as the experimental site for creation of full-thickness chondral defects followed by *LhCG* engraftment while the minor load-bearing regions located on the femoral trochlear groove areas were adopted as the donor site for autologous chondrocytes. The study duration was deliberately designed to determine the suitability of *LhCG* for *in situ* cartilage repair studies with the knowledge that fibrotic scar tissue becomes detectable in the same amount of time^26^. Live magnetic resonance imaging (MRI) was performed at stipulated time-points to assess cartilage repair. At the study endpoint, gross examination and histological outcomes of the repair were assessed. Additionally, repair tissue was quantitatively analysed in terms of its biochemical composition and biomechanical properties, and compared to native articular cartilage.

## Results

### Preparation of autologous *LhCG* for engraftment

**Figure 1:**
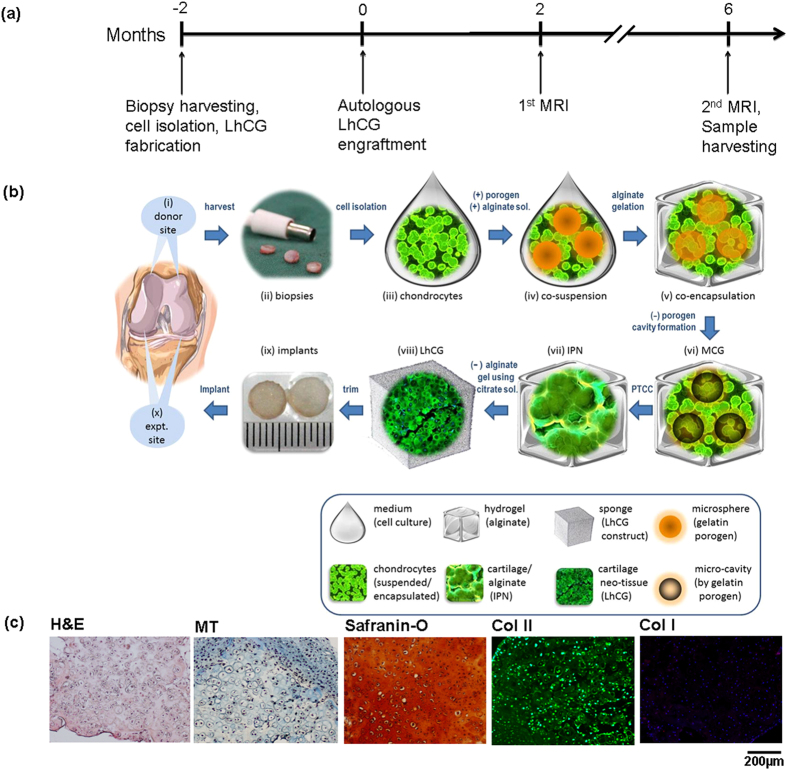
Experimental design (a) A timeline showing the various experimental steps of this study, spanning a total period of 8 months. (**b**) A schematic diagram showing the detailed process flow of this study. The study began with (i) identification of donor site, followed by (ii) biopsy harvesting from the minor load-bearing femoral trochlear groove regions, and (iii) chondrocytes extraction. After one passage (P1) of *in vitro* expansion, the chondrocytes were (iv) co-suspended and then (v) co-encapsulated with gelatin microspheres in alginate hydrogel. The gel constructs were cultured at 37 °C to dissolve the gelatin microspheres, resulting in micro-cavities formation. The resultant (vi) micro-cavitary hydrogel constructs (MCG) promoted vigorous cell proliferation and condensation into isogenic groups, eventually sprouting from the gel phase into the micro-cavities (PTCC) (21, 25). Upon prolonged culture, each neotissue aggregate that filled the micro-cavities eventually connected to one another, resulting in (vii) a 3D interpenetrating network (IPN). Following alginate gel removal, *LhCG* was generated in (viii). The *LhCG*s were then trimmed and implanted (ix) into critical-sized defects on the femoral condyles (indicated as expt. site in (x)). (**c**) Histological evaluation of the autologous *LhCG*s, showing the typical rounded morphology of chondrocytes, as well as their GAG and type II collagen-rich ECM (scale bar: 200 μm).

The preparation of autologous *LhCG* for engraftment into the load-bearing regions of the knee joints was performed according to the timeline presented in Fig. 1(a). The whole process began with harvesting articular cartilage biopsies from the minor load-bearing femoral trochlear groove regions, from which autologous chondrocytes were then extracted (Fig. 1(b)i–iii). To allow spontaneous healing, the defect size at these autologous donor sites (*ADS*) was 4 mm in diameter, which is below the reported critical sized defect of 6 mm in adult pigs^27^. Upon cell extraction and one passage (P1) of *in vitro* expansion, the chondrocytes were then used to construct *LhCG*s. The protocol for *LhCG* preparation has been established in our previous work^24^ and is depicted in Fig. 1(b)iv–viii. Briefly, autologous chondrocytes (P1) were co-suspended and then co-encapsulated with gelatin microspheres (~200 μm in diameter) in alginate hydrogel. When the gel constructs were cultured at 37 °C, the gelatin microspheres rapidly dissolved and leached out of the constructs, leaving behind micro-cavities of the same size throughout the cell-laden gel phase. These resultant micro-cavitary hydrogel constructs (MCG) allow cells to proliferate vigorously and condense into isogenic groups, eventually sprouting from the gel phase into the micro-cavities (Phase Transfer Cell Culture or PTCC)^24^,^28^. Following prolonged culture, the micro-cavities were filled with cartilaginous neotissue aggregates formed by the outgrowing chondrocyte isogenic groups. The aggregates in each micro-cavity eventually connected to one another, resulting in a 3D interpenetrating network (IPN) of the neotissue and the alginate gel phase. Upon complete removal of the alginate gel with citrate buffer, *LhCG* was generated. The whole process lasted two months, which is inclusive of the initial monolayer cell expansion (7~10 days), PTCC in MCGs (35 days) and the final maturation phase of *LhCG* (10~14 days).

Prior to engraftment, autologous *LhCG*s were evaluated histologically to confirm their hyaline-like phenotype. As seen in Fig. 1(c), the staining results demonstrated that the chondrocytes maintained their typical rounded morphology and were surrounded by abundant ECM (as seen in the Hematoxylin and Eosin (H&E) and Masson’s Trichrome (MT) images), which consists of glycosaminoglycan (GAG) (indicated by Safranin-O staining) and type II collagen (stained green). The presence of type I collagen, a fibrocartilage-related marker, remained undetectable. The results suggested that the autologous *LhCG*s produced were of hyaline-like phenotype. The engraftment surgery for autologous *LhCG*s was performed after two months, calculated from the point of articular cartilage biopsies harvesting to *LhCG* maturation. During the surgery, full-thickness critical-size defects (6 mm in diameter) were created on the femoral condyles. The autologous *LhCG*s were then trimmed to be press-fitted into the freshly created defects (Fig. 1(b)ix–x). These *LhCG*-engrafted sites were labelled as *LhCG*+. In parallel, 6 mm defects that were kept empty were used as negative controls and labelled as *LhCG*−.

### Live MRI monitoring of articular cartilage repair in mini-pigs

**Figure 2:**
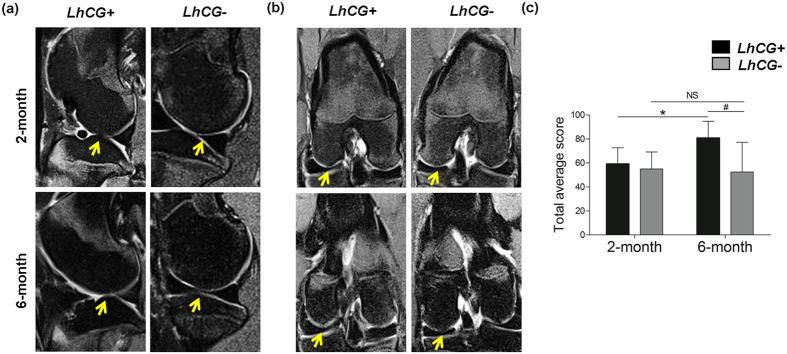
MRI and clinical evaluation of articular cartilage repair. Proton-density weighted MRI images in the (**a**) sagittal plane and (**b**) coronal plane for *LhCG*+ and *LhCG*− group at two months (baseline) and six months show progressive tissue repair after *LhCG* engraftment into the full-thickness chondral defects located at the major load-bearing regions of the knee (condyles). Arrows indicate the defect sites. (**c**) The total average scores were derived from (**a,b**) for both experimental groups obtained using the 2D-MOCART scoring system. A high score (min. 0; max. 100) indicates native-like cartilage tissue. Two-tailed, paired t-test was used for data analysis to compare scores at 2 months and 6 months within the same group (indicated by *p < 0.05) while two-tailed, unpaired t-test was used to compare scores between the *LhCG*+ group and *LhCG*− group (indicated by #, p < 0.05). Data are presented in the form of mean ± SD, with N = 5. Abbreviations: *LhCG*+, *LhCG*-repaired cartilage; *LhCG*−, self-repaired cartilage.

In order to monitor the progress of repair in a non-invasive manner, magnetic resonance imaging (MRI) was performed at 2-month and 6-month post-engraftment (Fig. 2). Subsequently, the MRI images were scored using the 2D MOCART scoring system with the parameters listed in Supplementary Table 1. At 2 months, the *LhCG*+ group performed comparably to the *LhCG*− group. However, at 6 months, the *LhCG*+ group showed a significant improvement (p value < 0.05) in the total score as compared to its baseline score at 2 months (Fig. 2(c)). More importantly, the radiographic score of *LhCG*+ was significantly higher than *LhCG*− (p < 0.05). *LhCG*+ out-performed *LhCG*− in terms of cartilage resurfacing, where 4 out of the 6 surfaces were restored in the former while all surfaces were damaged in the latter. Furthermore, 5 out of the 6 of the repaired tissue in the *LhCG*+ group had a homogeneous structure while all self-repaired tissue in the *LhCG*− group showed inhomogeneous structure or cleft formation.

### Macroscopic examination

**Figure 3:**
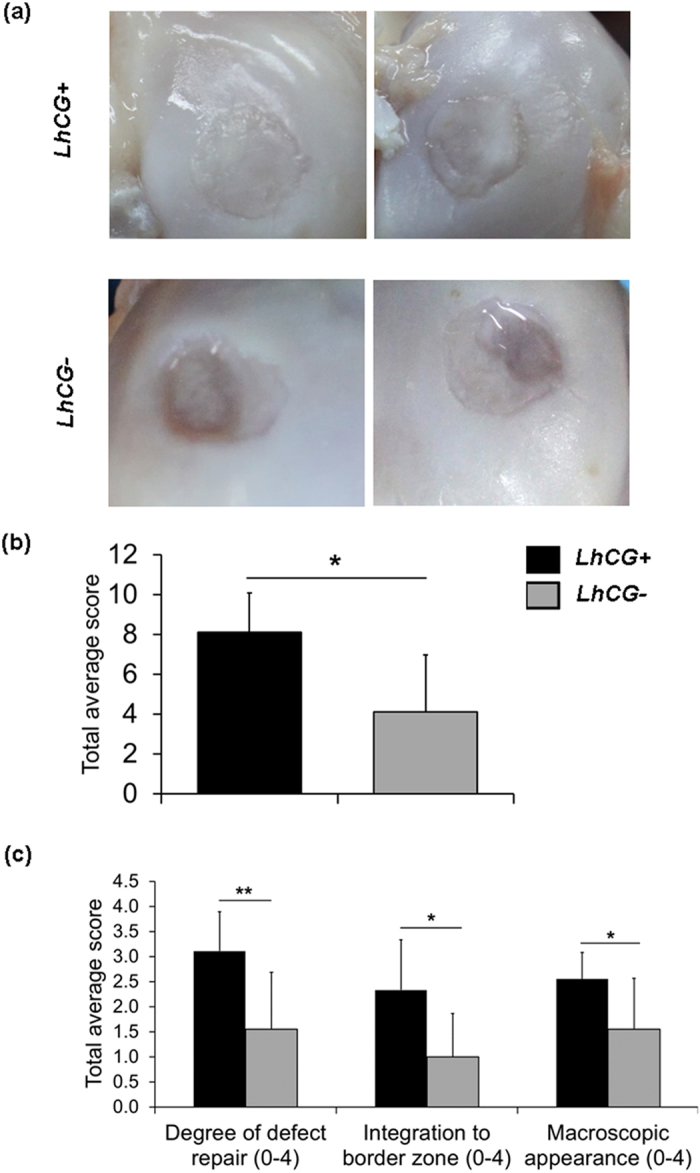
Macroscopic evaluation of cartilage repair at 6 months post-engraftment. (**a**) Representative photographs showing the macroscopic repair outcome. Full-thickness defects in the *LhCG*+ group (Top row) were filled with glossy white tissue while defects in the *LhCG*− group (Bottom row) remained insufficiently filled. (**b**) ICRS macroscopic mean scores for the two experimental groups. A high score (min. 0; max. 12) indicates native-like cartilage tissue. (**c**) The scores for the individual parameters in the ICRS scoring system. Nonparametric Mann Whitney test was used for data analysis. (*) indicates statistical significance difference (p < 0.05); (**) (p < 0.01). Data are presented in the form of mean ± SD, with N = 5.

Upon completion of the study at 6 months post-engraftment, the animals were sacrificed and their knees were excised and examined. In the *LhCG*+ group, full-thickness defects were repaired with glossy white tissue, which was comparable to normal articular cartilage in appearance (Top row, Fig. 3(a)). Contrastingly, defects in the *LhCG*− group remained largely unfilled (Bottom row, Fig. 3 (a)). The average ICRS (International Cartilage Repair Society) macroscopic score (as demonstrated in Supplementary Table 2)^29^ of the *LhCG*+ group (8.11 ± 1.96) was significantly higher than that of the *LhCG*− group (4.11±2.85) (Fig. 3(b), p < 0.05). The individual breakdowns of the score are shown in Fig. 3(c). Mean scores for the *LhCG*+ group in terms of defect filling (p < 0.01), integration to surrounding host cartilage (p < 0.05), and macroscopic appearance (p < 0.05) were all significantly higher than those of the *LhCG*− group. In line with the MRI results, macroscopic evaluations also suggested positive repair in the *LhCG*+ group while such observations were lacking in the *LhCG*− group.

### Histological and immunohistochemical evaluation

**Figure 4:**
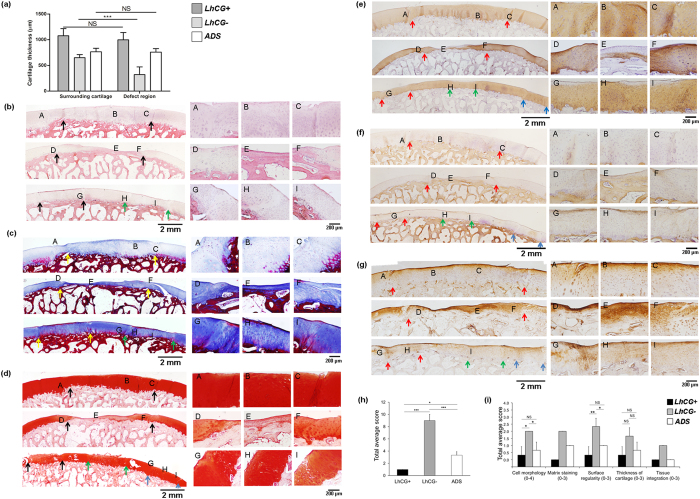
Histological and immunohistochemical analyses of the repaired tissue in the *LhCG*+*,LhCG*− and *ADS* groups. (**a**) The average cartilage thickness for each group was determined at the defect regions and their surrounding, unaffected regions. Two-tailed, unpaired t-test was used for data analysis. Evaluation of the repaired cartilage phenotype was based on: (**b**) H&E; (**c**) Masson’s Trichrome; (**d**) Safranin-O; (**e**) immunohistochemical staining for type II collagen; (**f**) immunohistochemical staining for type I collagen; (**g**) immunohistochemical staining for type VI collagen ((**A–I**) showed higher-magnification images at each specified location). Long scale bar: 2 mm; short scale bar: 200 μm. (**h**) The tallied histological scores based on the Wakitani scoring system. A low score (min. 0; max. 16) defines a native-like cartilage tissue with good defect filling and good integration with adjacent host cartilage. (i) Total average score for each specific parameter that contributed to cartilage restoration. The *LhCG*+ group (Upper panels in (**b–f**)) was repaired with hyaline-like tissue, which integrated well with adjacent host cartilage. The untreated group, *LhCG*− (Middle panels in (**b–f**)) was filled with irregular fibrocartilaginous tissue, which stained positively for type I collagen but not for type II collagen. The defects in the *ADS* (Lower panels in (**b–f**)) group self-repaired and showed no signs of degeneration. (*) indicates statistical significance (p < 0.05); (***) (p < 0.001). Each set of colored arrows demarcates individual defect sites. Data are presented in the form of mean ± SD, with N = 5.

In order to identify the quality of the repaired tissue, *LhCG*+, *LhCG*−, and *ADS* group were histologically evaluated. Using the histological images, the average cartilage thickness for each group was determined at the defect regions and their surrounding, unaffected regions. As presented in Fig. 4(a), the thickness of the *LhCG*+ group was found to be the closest to that of its surrounding cartilage and also the highest among all groups. Similar results were obtained for the *ADS* group. In contrast, the thickness value measured from the *LhCG*− group was found to be significantly lower than its surrounding tissue, particularly at the central regions of the defects (p < 0.001).

As shown in (Fig. 4(b–g), upper panel, centered at location (B)), the repaired cartilage generated in the *LhCG*+ group displayed tissue morphology similar to normal hyaline cartilage. At higher magnification, the cells appeared like well-differentiated chondrocytes and were surrounded by a matrix that is comparable to those of the adjacent host cartilage. More specifically, glycosaminoglycans (GAG, Safranin-O stains) and type II collagen (Fig. 4(d,e), upper panel, centered at location (B)) were evident throughout the tissue while type I collagen (Fig. 4(f), upper panel, centered at location (B)) was minimal. Type VI collagen was also present in a zone-specific manner, with no increased expression observed in the middle and deep zones (Fig. 4(g), upper panel, centered at location (B)). In contrast, untreated defects in the *LhCG*− group (Fig. 4(b–f) (middle panel, centered at location (E)) were filled mostly with disorganized fibrocartilage that did not restore a continuous articular surface with adjacent host cartilage. Furthermore, there was reduced staining for GAG (Fig. 4(d), middle panel, centered at location (E)) and type II collagen (Fig. 4(e), middle panel, centered at location (E)) while type I and type VI collagen staining was intense (Fig. 4(f,g), middle panel, centered at location (E)), suggesting that the self-repaired tissue was not healthy hyaline cartilage.

In terms of cellular organization, the *LhCG*+ group showed a greater extent of columnar organization than the *LhCG*− group. Moreover, repaired cartilage generated in the *LhCG*+ group was also observed to integrate well with the surrounding cartilage without gaps or discontinuities. As far as the *ADS* group is concerned (Fig. 4(b,f), lower panel, centered at location H), in which the defects were created for autologous cell extraction and left untreated for eight months, the defects showed significantly better self-repair as compared to the *LhCG*− group. Upon closer examination, the *ADS* group showed a more fibrous structure in the matrix and reduced staining for GAG as compared to the surrounding, unaffected cartilage (Fig. 4(c,d), lower panel, centered at location H). Additionally, the immunostaining results indicated that type I, II and VI collagen were present in deeper parts of the self-repaired tissue. At the articulating surface, however, type I collagen was particularly abundant while type II collagen was minimally present (Fig. 4(e,f), lower panel, centered at location H). All these observations suggested that fibrocartilage was predominantly lining the surface of the self-repaired tissue while hyaline-like cartilaginous tissue was generated in deeper parts.

Based on the histological and immunohistochemical staining outcome, mean histological scores for all three groups were obtained using Wakitani scoring system (as demonstrated in Supplementary Table 3)^30^. As presented in Fig. 4(h), the overall mean score for the *LhCG*+ group (1, no standard deviation) was significantly lower than that of the *LhCG*− (9 ± 1) and the *ADS* group (3±0.58), indicating a hyaline-like repair. The scores for five key parameters of chondral restoration, which contribute to the overall histological scores of each sample, were displayed in Fig. 4(i). The parameters that distinguished the *LhCG*+ group from the other two groups were: tissue morphology, matrix staining, and tissue integration.

Besides compositional analysis of the matrix, a more detailed analysis was also performed to evaluate the ultrastructure of the repaired tissue generated in all groups. Tissue ultrastructure is mainly reflected by the orientation and arrangement of collagen fibres, which was visualized via picrosirius polarization. In general, thin collagen fibers or poorly packed collagen display green to greenish yellow polarizing colors, whereas thick or tightly packed fibers show yellowish-orange through orange to red polarizing colors^31^. In addition, type I collagen may show up as thick (2–10 μm in diameter), strongly birefringent, yellow or red fibers whereas type II collagen forms fibrils (20–30 nm in diameter) and shows weak birefringence of a varying color^32^.

**Figure 5:**
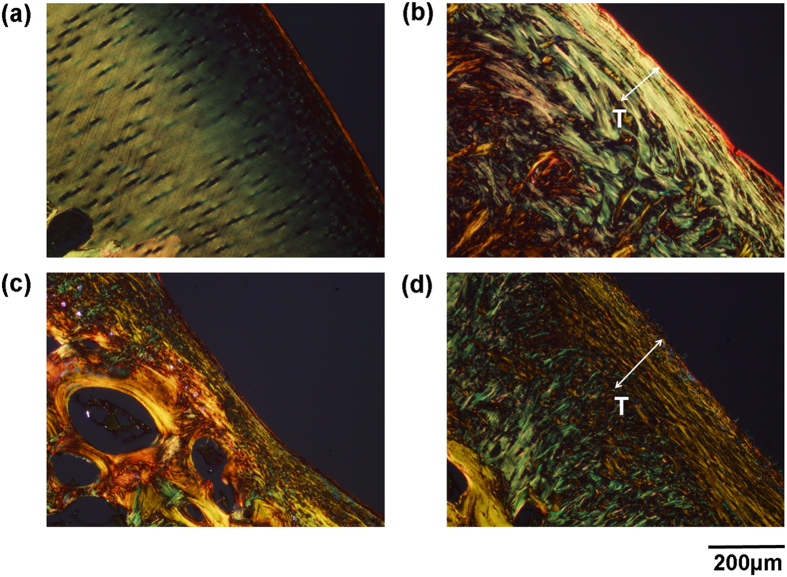
Evaluation of tissue ultrastructure via picrosirius polarization method. The orientation and arrangement of collagen fibers/fibrils as observed in the matrix of the (**a**) native articular cartilage; (**b**) *LhCG*+; (**c**) *LhCG*−; (**d**) *ADS* groups. Generally, green to greenish yellow represents thin or poorly packed collagen fibrils while yellowish-orange through orange to red represents thick or tightly packed collagen fibers. Scale bar: 200 μm.

From the orientation of the collagen (Fig. 5), the superficial tangential zone (STZ, indicated as T) was clearly delineated in the *LhCG*+ group (Fig. 5(b)), but not in the *LhCG*− group (Fig. 5(c)). From the perspective of collagen arrangement, *LhCG*+ group displayed predominantly thin, loose fibrils that are representative of type II collagen. On the other hand, *LhCG*− group predominantly exhibited thick, packed fibers that indicate type I collagen. In the *ADS* group, the STZ comprised of thick, tight type I collagen fibers, while the deeper zones exhibited thin, loose type II collagen fibrils.

### Biochemical and biomechanical analyses

**Figure 6:**
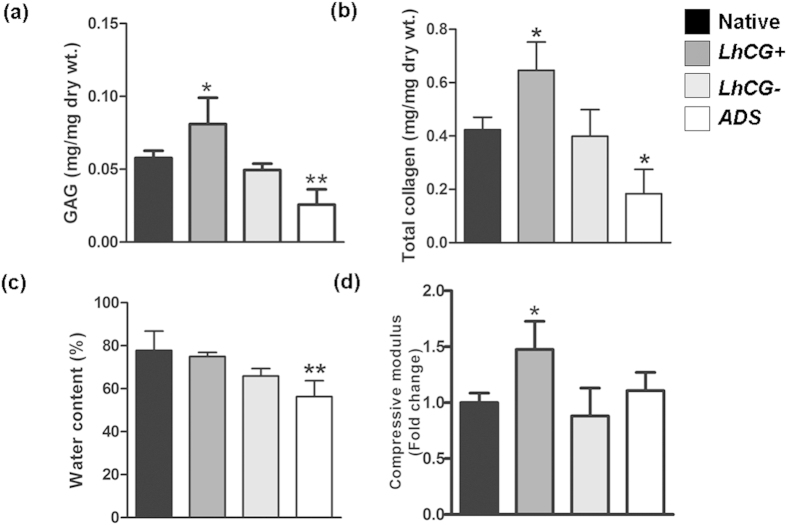
Quantitative evaluation of articular cartilage repair. Biochemical analysis was performed to quantify the (**a**) GAG content and (**b**) total collagen content in all the explanted tissues. To correlate biochemical aspects with the functional aspects of the tissue, (**c**) water content and (**d**) compressive moduli were also determined. The results for the three experimental groups, namely *LhCG*+*, LhCG*− and *ADS* group were compared to native, unaffected cartilage. One-way ANOVA/Dunnett’s multiple comparison test was used for data analysis. (NS) indicates no statistical significance between two groups while (*) indicates statistical significance (p < 0.05); (**) (p < 0.01). Data are presented in the form of mean ± SD, with N = 5.

Biochemical and biomechanical properties of articular cartilage tissue are closely linked^1^. As indicated in Fig. 6(a), GAG content of the *LhCG*+ group was significantly higher (*, p < 0.05) than native cartilage, whereas the *LhCG*− (NS, p > 0.05) and *ADS* groups (**, p < 0.01) exhibited lower GAG contents. When assessed for their total collagen content (Fig. 6(b)), only the *LhCG*+ group was found to be significantly higher than the native cartilage (p < 0.05). The water content results revealed that only *ADS* group possessed significantly lower water content than that of native cartilage (Fig. 6(c), p < 0.01). The mechanical (compressive) results showed that *LhCG*+ group had significantly higher compressive modulus than all other groups (Fig. 6(d)), (p < 0.05).

## Discussion

The capacity of cartilage to self-heal after injury is known to be very limited, predisposing it to downstream issues like OA. Despite the improvement in technology and the birth of tissue engineering in the early 90 s, an effective treatment for articular cartilage repair remains elusive^16^. Among the currently available techniques mentioned earlier in this study, ACI comes across as a cell-based tissue engineering approach for articular cartilage repair. Its technique involves 2D culture expansion of chondrocytes, which may alter their phenotype. On this note, there are studies that suggest that a 3D environment may help to maintain the original cellular phenotype^33^,^34^. With this in mind, the ACI evolved to employ 3D scaffolds to encapsulate and deliver chondrocytes, a technique known as MACI^35^. In general, the ACI/MACI treatment procedure consists of three main steps: (1) harvesting of autologous cartilage from minor load-bearing sites via minimally invasive surgery; (2) *in vitro* extraction and culture expansion of chondrocytes; and (3) re-implantation of the chondrocytes into the defect either with or without scaffolds (in 3D scaffolds for MACI)^35^.

In the current study, harvesting of autologous cartilage was similarly done in minor load-bearing sites, with minimal donor site morbidity as evidenced by histological, biochemical, and biomechanical results (Figs 4 and 6) of the *ADS* samples. This finding was comparable to step (1) of the ACI approach. In order to obtain a sufficient number of cells for treatment, the ACI approach requires extensive cell expansion in monolayer culture (step (2)), which inevitably causes phenotypic alterations of chondrocytes^36^. Contrastingly, the chondrocytes used for *LhCG* fabrication experienced only one passage of monolayer expansion followed by a 35-day culture (PTCC) in 3D alginate-based MCG constructs (Fig. 1 (b) Step iii~viii)^22^. This strategy allows cells to maintain their differentiated phenotype, as shown through the retention of their characteristic rounded morphology and capacity to synthesize cartilage-specific molecules, which is in agreement with other reports^37^,^38^,^39^. Additionally, prolonged culture expansion in a non-cell adhesive 3D environment also helps in removing adherent cell types (e.g. osteoblasts), which may be unintentionally harvested from the subchondral bone region. This purifies the isolated cell population for non-anchorage dependent cells like the chondrocytes^40^.

Following the PTCC process, chondrocytes were pre-formed into centimeter-sized *LhCG* constructs with a defined shape and structure. The biological resemblance of the constructs to native articular cartilage was demonstrated in Fig. 1 (c). The presence of abundant type II collagen and GAG is important as they have been reported to correlate with the mechanical quality of engineered hyaline cartilage^41^. This strategy of re-implanting an engineered living tissue instead of dissociated cells (as per 1^st^ and 2^nd^ generation ACI approach, step (3)), circumvents issues regarding cell retention and a non-homogeneous cell distribution at the recipient site^15^. In comparison to the third generation of ACI, which involves the use of scaffolds in step (3), the *LhCG* is scaffold-free, and the adoption of this approach avoids the contamination of its purity by any exogenous materials. Furthermore, from a biological perspective, the purity of both *LhCG* and ACI is also enhanced by the use of a homogeneous population of autologous chondrocytes. In contrast to stem/progenitor cells, chondrocytes are more attractive since no further manipulation is required (such as providing the appropriate growth factors, signaling molecules, or physical influences for precise control of differentiation)^42^.

Besides the advantages of the *LhCG* platform in cell expansion and maintaining cartilage phenotype *in vitro*, it is also important to address two key requirements in *in vivo* cartilage repair^43^: to restore the articular surface with repair tissue that has similar biological and biomechanical properties as articular cartilage; and to promote successful integration of the repair tissue with the adjacent native articular cartilage. To investigate the capacity of *LhCG*s in fulfilling these requirements, they were implanted into critical sized defects created on the femoral condyles. The *in vivo* results after six months were promising. From a radiographic perspective (MRI), the *LhCG*+ group was observed to restore the articular cartilage, as compared to the *LhCG*− group (Fig. 2). At the study endpoint, macroscopic observations presented similar findings, wherein the *LhCG*+ group achieved most complete filling of defect as compared to the *LhCG*− group. Scoring of macroscopic repair corroborated well with these observations, with the degree of repair and integration to host cartilage in *LhCG*+ out-performing *LhCG*− (p < 0.01) (Fig. 3).

Upon histological analysis of tissue harvested at 6 months post-engraftment (Fig. 4), hyaline-like cartilaginous tissue formation was apparent in the *LhCG*+ group, as evidenced by the presence of lacunae and cells that were embedded within basophilic ground substance that was rich in GAG and type II collagen. In the case of the *LhCG*− group, fibro-cartilaginous repair tissue was observed, as indicated by the presence of more type I collagen while less proteoglycans were present as compared to native articular cartilage. Besides the major ECM components, the expression of type VI collagen was also investigated in all three experimental groups. This minor protein seems to play an important role for the integrity of the cartilage matrix by anchoring chondrocytes to their surrounding matrix^44^. Under normal conditions, this protein occurs primarily in the pericellular matrix that surrounds the chondrocytes throughout the different zones. In OA cartilage, it was reported that the expression and overall distribution of type VI collagen increases in the lower middle and upper deep zones^45^,^46^. In the results presented in Fig. 4(g), it could be observed that the lower middle and upper deep zones of *LhCG* repaired cartilage were morphologically similar, with no particular increased expression of this protein. On the other hand, *LhCG*− (self-repaired cartilage) showed intense staining in the same regions. Collectively, the results suggested that *LhCG* repaired cartilage did not exhibit signs of cartilage degeneration.

As mentioned in the preceding text, the use of autologous cell source is ideal to enhance clinical translation, but it raises concerns about donor site morbidity^47^. Therefore, in addition to the two experimental groups (*LhCG*+ and *LhCG*−), the donor site conditions (*ADS*) at endpoint were also deliberately assessed. The histology results for the ADS group showed that the defects located at minor-load bearing areas were capable of self-repair, as seen by obvious wound healing and minimal scar tissue formation. Taken together, these results suggested that *LhCG* engraftment was able to withstand load bearing and had repaired the defect site with cartilaginous tissue that exhibits similar characteristics to native hyaline cartilage. Thus far, a major challenge has been addressed^48^,^49^,^50^, which is in maintaining hyaline-like phenotype post-implantation; the lack of a true hyaline quality of the repaired cartilage tissue escalates the development of OA.

After being shown to produce repaired cartilage with hyaline-like phenotype, the ability of *LhCG* to integrate with the host articular cartilage was also evaluated. Poor integration has traditionally plagued many cartilage repair approaches due to the low metabolism of cartilage and its dense, anti-adhesive ECM^16^,^51^. In the case of the *LhCG*+ group, the ECM produced by *LhCG* integrated into the surrounding cartilage resulting in significantly better macroscopic and microscopic healing of the cartilage defects when compared to untreated controls (*LhCG*−). No signs of cleft, delamination, and fissuring were observed at the boundary between host and repair tissue (Fig. 4(b–f)). As suggested before, this can be attributed to the sponge-like properties of *LhCG*^24^. Its lower mechanical stiffness compare to native cartilage enabled *in situ* remodeling and maturation, resulting in good integration with surrounding native cartilage. Further benefit of host-implant integration was manifested in terms of cartilage thickness restoration. In the *LhCG*+ group, cartilage thickness of the defect regions and also of the adjacent host cartilage regions was thicker than those in the *LhCG*− and *ADS* groups (Fig. 4(a)). This was due to more uniform load distribution arising from host-implant integration^16^. In summary, these results demonstrated that the *LhCG* was able to promote host-implant integration.

It is well established that the mechanical properties of articular cartilage are closely related to the two main components of the hyaline cartilage matrix, namely the solid macromolecular framework (comprised of chiefly collagens and aggregating proteoglycans), and the water content within this framework^52^. Hence, the biochemical make-up and biomechanical responses of explanted tissues were determined at endpoint (Fig. 6) to evaluate cartilage regeneration in more detail. According to Buckwalter and Mankin, type II, IX, and XI collagens are responsible for the form, tensile stiffness, and strength of cartilage tissue, while large aggregating proteoglycans are responsible for its compressive stiffness^1^. The results in this study are in agreement with their statement, where the higher content of GAG in the *LhCG*+ group conferred a higher compressive modulus. Interestingly, both GAG and total collagen content of *LhCG*+ were significantly higher than native cartilage. It is postulated that this increase in matrix synthesis was a combined effect of the innate response of chondrocytes to injuries and *LhCG* engraftment. An injury to the articular surface usually triggers chondrocytes in the vicinity to proliferate and increase synthesis of matrix macromolecules. However, this anabolic response of chondrocytes ceases soon after injury, resulting in incomplete filling of tissue defect^53^,^54^. The presence of *LhCG* might have supported and prolonged the compensatory mechanisms of chondrocytes, leading to overproduction of GAG and collagens. This consequently results in the significantly higher compressive modulus than native cartilage.

Further investigation was carried out on tissue ultra-structure, which also plays an integral role in the regenerative process by transferring compressive loads from the directly loaded regions to the adjacent zones. In particular, Hosseini *et al.*^55^ reported that the STZ is very important for this purpose. The STZ restored by *LhCG* was continuous and well defined, as observed through the picrosirius red stain (Fig. 5(b)). Closer observations revealed that the collagen fibrils superficial to the articular surface were arranged in a tangential direction, before subsequently being arranged transversely. This hierarchy of structures was previously reported by Kaab *et al.*^56^ and was observed in our study, in the native cartilage and *LhCG*+ groups. The observable difference between native cartilage and the *LhCG*+ group lies in the depth of the tangential region, where it was found to be much thicker in the latter. On this note, we believe with progressive remodeling of the repaired cartilage, the STZ may eventually become thinner when the deep zone of the cartilage is fully regenerated. According to the recommendations of ICRS, a longer recovery period is required for tissue structure to be regained, and this duration can be up to 12 months^57^.

Among the various techniques available, scaffold-free techniques have gained interest in cartilage tissue engineering because they are able to mitigate the some of the challenges related to the use of scaffolds such as toxicity of degradation byproducts, stress-shielding, phenotypic changes, and potential scaffold interference with cell-cell communication^21^,^58^,^59^. Recently, layered chondrocyte sheets have been used to treat full-thickness cartilage defects in mini-pigs^48^. Good defect filling and integration were reported; however, in contrast to *LhCG* engraftment of this study, predominant expression of type I collagen was observed in the repaired cartilage. In addition, the surgical procedures for *LhCG* engraftment are relatively simpler since it can be press-fitted into the defects; no suturing was needed for fixation. Having shown that *LhCG* survived the load-bearing conditions *in vivo* in this study, longer-term studies (12–18 months^26^) are warranted to evaluate the durability of *LhCG*-mediated repair. Lastly, it should be noted that the mini-pigs in this study returned to full activity immediately after the implantation surgery. In the clinic, however, the human patients are advised against engaging in strenuous activities in the early days/weeks after surgery, followed by a gradual rehabilitation process to improve load-bearing on their joints to provide appropriate mechanical stimuli^60^,^61^. The same routine may be applicable for future *LhCG* studies in preclinical models, in order to minimize/avoid the induction of post-traumatic OA.

In summary, this study has demonstrated that the *LhCG* platform may be extended to autologous chondrocytes originated from mature cartilage, with encouraging cartilage repair observed after six months in a skeletally mature mini-pig model. Particularly, the *LhCG* exhibited macroscopic repair and integration with host cartilage, manifested in the maintenance of cartilage thickness and the restoration of an intact articular surface. More importantly, the reparative tissue generated by *LhCG* was biologically comparable to native hyaline cartilage, suggesting the potential to result in a more durable repair. This technique may have potential clinical use as the retrieval of cartilage explants may be performed arthroscopically, and the entire PTCC process may be conducted in a GLP facility. These encouraging results and clinically relevant surgical techniques potentiate the development of *LhCG* beyond a preclinical setting.

## Methods

### Experimental design and study population

Animal surgeries were performed in line with the guidelines for the care and use of laboratory animals, and the protocols were approved by the Institutional Animal Care and Use Committee (IACUC). Five female, skeletally matured mini-pigs (15-17 months of age; mean body weight, 29 kg) were used for this study. They were supplied by at PWG Genetics Pte Ltd (GLP certified contract research organization), Singapore and were maintained under specific-pathogen-free conditions. Two surgeries were performed: the first surgery was for cartilage biopsy harvesting and the second surgery for *LhCG* engraftment was performed two months later. Sample size was chosen based on an estimate of 5 samples per test group, without prior statistical analysis. Randomization was not used in this study.

### Cartilage biopsy harvesting

Small cartilage discs (eight per knee, each with a diameter of 4 mm) were harvested for cell isolation and expansion from a minor load-bearing region of the host medial and lateral upper femoral trochlear area using a biopsy punch.

### Autologous chondrocytes isolation and *in vitro* expansion

*Unless stated otherwise, all cell-culture reagents were supplied by Invitrogen, Life technologies.* Approximately 300–400 mg of cartilage discs were harvested from each mini-pig. Subsequently, the cartilage discs were rinsed in sterile Phosphate Buffered Saline (PBS), followed by thorough washing in penicillin (100 units/mL) and streptomycin (100 mg/ mL). The discs were then minced into fragments using tweezers and surgical blades, and subsequently digested in culture media containing collagenase II at a concentration of 1 mg/mL. Following enzymatic digestion, isolated chondrocytes were plated according to each pig in their designated tissue culture flasks, and cultured in Dulbecco’s Modified Eagle Medium (DMEM) supplemented with 10% fetal bovine serum (FBS) and penicillin (100 units /mL) and streptomycin (100 mg/mL).

### Autologous Living Hyaline Cartilaginous Graft (*LhCG*) preparation

*Unless specifically mentioned, all chemicals used were purchased from Sigma Aldrich, Singapore while all cell-culture reagents were purchased from Invitrogen, Life Technologies.* The fabrication and characterization of *LhCG* were previously conducted and a similar protocol was used this study^24^. Briefly, gelatin microspheres with a porogenic role were fabricated using a oil-in-water-in-oil double-emulsion system and sieved into a size range of 150 to 180 μm. Subsequently, the microspheres were sterilized in PBS containing 1000 units/mL of penicillin and 1000 mg/mL of streptomycin overnight and stored in sterile PBS at 4 °C. Upon reaching 80% confluence, autologous chondrocytes (described in previous section) were individually trypisinized, washed and centrifuged before being co-suspended (1 × 10^7^ cells/mL) with gelatin micropsheres in chilled alginate solution (1.5% w/v in 0.15 mM aqueous calcium chloride was added gently for gelation. Each individually matched *LhCG* was then trimmed into dimensions of circa 6 mm × 5 mm × 1.5 mm and cultured on agarose gel-coated tissue culture plates in chondrocyte culture (CC) medium that composed of Dulbecco’s modified Eagle medium (DMEM) supplemented with 20% (v/v) fetal bovine serum (FBS, Invitrogen), 4-(2-hydroxyethyl)-piperazine-1-ethanesulfonic acid (HEPES, 0.01 M), non-essential amino acids (NEAA, 0.1 mM), proline (0.4 mM), vitamin C (0.05 mg/mL), penicillin (100 units/mL), and streptomycin (100 mg/mL). The *LhCG*s were maintained on an orbital shaker at 50 rpm in a humidified incubator at 37 °C with 5% CO_2_. Upon reaching maturity after 35 days, the *LhCG*s were immersed in sodium citrate (55 mM in 0.15 M NaCl) to remove the alginate bulk (10 mins, at room temperature) and were cultured for another ten days prior to implantation.

### Autologous *LhCG* engraftment into full-thickness chondral defects

All surgeries were performed under general anaesthesia, which was induced by intramuscular administration of Atropine (0,05 mg/kg) followed by xylazil (2.5 mg/kg) and ketamine (10 mg/kg). Additionally, the animals were intubated using an appropriate size cuffed endotracheal tube and anaesthesia was maintained by inhalation of 1–3% of isoflurane. Full thickness chondral defects of 6.0–6.3 mm in diameter and 1.5–2.0 mm in depth were created on both condyles using biopsy punches. *LhCG*s were punched out using the same biopsy punches and were press-fitted into the defect either on the medial or lateral condyles (arbitrarily assigned) while the other defect was left empty. This interference fit, coupled with the nature of *LhCG*, which is sponge-like, no further fixation was needed to secure the graft. The mini-pigs were allowed to move freely after the surgery. The abbreviations for the three experimental groups are as follows: *LhCG*-repaired tissue (*LhCG*+), untreated control (*LhCG*−), and donor site for autologous cells (*ADS*).

### Magnetic Resonance Imaging (MRI)

The mini-pigs were imaged under general anesthesia at 2 months and 6 months post-surgery. MRI images of the entire knee joints were obtained using an 18-channel body matrix coil on a clinical 3T MRI Scanner (Siemens, Skyra). A fat-saturated proton density-weighted MR imaging sequence was used, without any contrast agent. All three planes were acquired. Imaging parameters were as follows: repetition time (TR) = 3800 ms, echo time (TE) = 39 ms, field of view (FOV) = 130 mm, matrix size = 384 × 384, slice thickness = 2 mm, slice gap = 0.2 mm. MRI images were independently scored by an experienced radiologist (blinded), specialized in musculoskeletal imaging, using a modified clinical 2D MOCART scoring system^62^,^63^ (Supplementary Table 1).

### Implanted tissue harvesting

Six months following engraftment, the mini-pigs were sacrificed by intravenous injection of pentabarbital (150 mg/kg). The entire knee joints were collected, exposed, and photographed. A surgical saw was used to collect an area of surrounding host tissue containing the implant and underlying bone from each defect site.

### Macroscopic examination

The repaired cartilage was examined macroscopically and scored using International Cartilage Repair Society (ICRS) scoring system for cartilage repair^29^ (Supplementary Table 2). After macroscopic evaluation, half of the samples were used for histological and immunohistochemical staining while the remaining samples were used for biomechanical testing followed by biochemical analysis.

### Histological evaluation

Collected samples were fixed in 10% buffered formalin and decalcified, followed by standard histological processing. Histology slides were stained with H&E, Safranin-O, Masson’s Trichrome, and picrosirius red according to standard protocols. Regenerated cartilage was scored using the scoring system described by Wakitani and colleagues^30^. (Supplementary Table 3) The criteria considered by this histological scoring system include cell morphology, matrix staining, surface regularity, cartilage thickness, and integration with host adjacent cartilage. Samples were scored independently by three evaluators who were blinded to experimental data. The scores of the three observers were averaged to obtain a representative score for each criterion.

### Immunohistochemical staining

For immunohistochemical detection of type I, II and VI collagen, tissue sections were deparaffinized, rehydrated and stained using UltraVision Quanto Detection System HRP DAB kit (Thermo Scientific) according to the manufacturer’s protocol. All primary antibodies were purchased from Abcam. For type II collagen staining, the subchondral bone was used as a negative control while the adjacent articular cartilage as a positive control since this collagen type is exclusively present in the articular cartilage. Likewise, the subchondral bone serves as a positive control for type I collagen staining while the surrounding native cartilage (hyaline cartilage has negligible amount of collagen type I) was treated as a negative control.

### Biomechanical Testing and Biochemical analyses

Native articular cartilage in the same weight-bearing region from mini-pigs served as positive controls in the biomechanical testing. For the analysis of biomechanical properties, the compressive moduli of the collected samples were measured as described by Cui *et al.* Briefly, 4 mm diameter *LhCG*-repaired tissue and auto-regenerated tissue were punched out for biomechanical analyses. A constant, compressive crosshead moving speed of 1 mm/min was applied until reaching the end-of-test criterion of 400 N. A force–displacement curve was then obtained. The compressive modulus of the tested tissue was automatically calculated by the auxiliary software in the equipment (Instron, USA).

Following biomechanical testing, the samples were subjected to biochemical analyses. Briefly, samples were frozen at −20 °C before freeze-drying for 24 h, followed by overnight digestion in papain buffer consisting of 0.3 mg/ml papain dissolved in 0.2 mM dithiothreitol and 0.1 mM disodium ethylene diamine tetraacetic acid. Chondrocyte density was extrapolated from DNA content measured by the fluorometric Hoechst 33258 assay. Glycosaminoglycan (GAG) content was measured by 1,9-dimethylmethylene blue (DMMB) dye binding assay while total collagen content was quantified by using proline/hydroxyproline assay from acid hydrolyzed samples.

### Statistical analysis

All data in this study are presented in the format: average ± standard deviation. Table 1 describes the statistical analyses used to determine statistical significance in this study (all at 95% confidence interval).

## Additional Information

**How to cite this article**: Peck, Y. *et al.* A preclinical evaluation of an autologous living hyaline-like cartilaginous graft for articular cartilage repair: a pilot study. *Sci. Rep.* **5**, 16225; doi: 10.1038/srep16225 (2015).

## Supplementary Material

Supplementary Information

## Figures and Tables

**Table 1:** Statistical analyses used to determine statistical significance in this study.

Statistical Test type/Post-hoc (if any)	Data set	Justification
Two-tailed, paired t-test	2D MOCART score	Compare scores of two dependent groups (post-defect creation (2 months) and post-regeneration (6 months)).
Nonparametric Mann Whitney test	Macroscopic repair score	Compare ICRS scores (non-Gaussian population) of two independent groups (*LhCG*+ and *LhCG*−).
Two-tailed, unpaired t-test	2D MOCART (at 6 months), cartilage thickness measurement	Compare radiographic scores between the *LhCG*+ group and *LhCG*− group at 6 months; compare cartilage thickness of the surrounding cartilage and the defect region)
One-way ANOVA/Dunnett’s Multiple Comparison Test	Biochemical and biomechanical analyses	Compare measurements of four unmatched groups (Native, *LhCG*+, *LhCG*−, *ADS*) with all treatment groups against the native cartilage.
