# Supplementary material for: A preclinical evaluation of an autologous living hyaline-like cartilaginous graft for articular cartilage repair: a pilot study
Source: Sci Rep. 2015 Nov 9;5:16225. doi: 10.1038/srep1622510.1038/srep16225 (PMC4637897; doi:10.1038/srep1622510.1038/srep16225)
Supplement: Supplementary Information [file srep16225-s1.pdf]

A preclinical evaluation of an autologous living hyaline-like cartilaginous graft for articular cartilage repair: a pilot study

Authors: Yvonne Peck[1], Pengfei He[1], Geetha Soujanya V.N. Chilla[1], Chueh Loo Poh[1], Dong-An Wang[1]*

Supplementary tables

**2D MOCART score**		
**Parameter**	**Item**	**Points**
Defect fill	Subchondral bone exposed	0
	Incomplete < 50%	5
	Incomplete > 50%	10
	Complete	20
	Hypertrophy	15
Cartilage interface	Complete	15
	Demarcating border visible	10
	Defect visible < 50%	5
	Defect visible > 50%	0
Surface	Surface intact	10
	Surface damaged < 50% of depth	5
	Surface damaged > 50% of depth	0
Adhesions	Yes	5
	No	0
Structure	Homogeneous	5
	Inhomogeneous or cleft formation	0
Signal intensity	Normal	30
	Nearly normal	10
	Abnormal	0
Subchondral lamina	Intact	5
	Not intact	0
Subchondral bone	Intact	5
	Granulation tissue, cyst, sclerosis	0
Effusion	No effusion	5
	Effusion	0
**Total points**		**100**

Supplementary Table 1. 2D MOCART evaluation of cartilage repair. Assessment of articular cartilage repair tissue in the femoral condyles by using the modified clinical 2D magnetic resonance observation of cartilage repair tissue (MOCART) score [62,63].

**Cartilage repair assessment ICRS**	**Points**
**Degree of defect repair**	
In level with surrounding cartilage	4
75% repair of defect depth	3
50% repair of defect depth	2
25% repair of defect depth	1
0% repair of defect depth	0
**Integration to border zone**	
Complete integration with surrounding cartilage	4
Demarcating border < 1 mm	3
3/4th of graft integrated,1/4th with a notable border > 1 mm width	2
1/2 of graft integrated with surrounding cartilage, 1/2 with a notable border > 1 mm	1
From no contact to 1/4th of graft integrated with surrounding cartilage	0
**Macroscopic appearance**	
Intact smooth surface	4
Fibrillated surface	3
Small<comma> scattered fissures or cracs	2
Several<comma> small or few but large fissures	1
Total degeneration of grafted area	0
**Overall repair assessment**	
Grade I: normal	12
Grade II: nearly normal	11-8
Grade III: abnormal	7-4
Grade IV: severely abnormal	3-1

Supplementary Table 2. ICRS Score. Assessment of macroscopic repair by using the International Cartilage Repair Society (ICRS) score for cartilage repair [29].

**Cell morphology**	
Hyaline cartilage	0
Mostly hyaline cartilage	1
Mostly fibrocartilage	2
Mostly non-cartilage	3
Non-cartilage only	4
**Matrix staining (metachromasia)**	
Normal (compared with host adjacent cartilage)	0
Slightly reduced	1
Markedly reduced	2
No metachromatic stain	3
**Surface regularity (total smooth area compared with entire area of cartilage defect)**	
Smooth (> 3/4)	0
Moderate (> 1/2–3/4)	1
Irregular (1/4–1/2)	2
Severely irregular (< 1/4)	3
**Thickness of cartilage (compared with that of surrounding cartilage)**	
>2/3	0
1/3–2/3	1
<1/3	2
**Integration of donor with host adjacent cartilage**	
Both edges integrated	0
One end intergrated	1
Neither edge integrated	2

Supplementary Table 3. Wakitani score. Histological evaluation of the cartilage repair tissue using a scoring system developed by Wakitani et al.[30].
